# Genetic insights into the globally invasive and taxonomically problematic tree genus *Prosopis*

**DOI:** 10.1093/aobpla/plaa069

**Published:** 2020-12-08

**Authors:** María L Castillo, Urs Schaffner, Brian W van Wilgen, Noé Manuel Montaño, Ramiro O Bustamante, Andrea Cosacov, Megan J Mathese, Johannes J Le Roux

**Affiliations:** 1 Centre for Invasion Biology, Department of Botany and Zoology, Stellenbosch University, Matieland, South Africa; 2 CABI, Rue de Grillons, Delémont, Switzerland; 3 Departamento de Biología, División de Ciencias Biológicas y de la Salud, Universidad Autónoma Metropolitana-Iztapalapa, CP, Mexico City, Mexico; 4 Departamento de Ciencias Ecológicas, Instituto de Ecología y Biodiversidad, Facultad de Ciencias, Universidad de Chile, CP, Santiago, Chile; 5 Laboratorio de Ecología Evolutiva - Biología Floral, Instituto Multidisciplinario de Biología Vegetal IMBIV, CONICET-Universidad Nacional de Córdoba, Argentina, CP, Córdoba, Argentina; 6 Department of Biological Sciences, Macquarie University, Sydney, NSW, Australia

**Keywords:** Eastern Africa, genetic diversity, hybridization, invasive alien species, mesquite, microsatellites, polyploidy, taxonomic uncertainty, tree invasions

## Abstract

Accurate taxonomic identification of alien species is crucial to detect new incursions, prevent or reduce the arrival of new invaders and implement management options such as biological control. Globally, the taxonomy of non-native *Prosopis* species is problematic due to misidentification and extensive hybridization. We performed a genetic analysis on several *Prosopis* species, and their putative hybrids, including both native and non-native populations, with a special focus on *Prosopis* invasions in Eastern Africa (Ethiopia, Kenya and Tanzania). We aimed to clarify the taxonomic placement of non-native populations and to infer the introduction histories of *Prosopis* in Eastern Africa. DNA sequencing data from nuclear and chloroplast markers showed high homology (almost 100 %) between most species analysed. Analyses based on seven nuclear microsatellites confirmed weak population genetic structure among *Prosopis* species. Hybrids and polyploid individuals were recorded in both native and non-native populations. Invasive genotypes of *Prosopis juliflora* in Kenya and Ethiopia could have a similar native Mexican origin, while Tanzanian genotypes likely are from a different source. Native Peruvian *Prosopis pallida* genotypes showed high similarity with non-invasive genotypes from Kenya. Levels of introduced genetic diversity, relative to native populations, suggest that multiple introductions of *P. juliflora* and *P. pallida* occurred in Eastern Africa. Polyploidy may explain the successful invasion of *P. juliflora* in Eastern Africa. The polyploid *P. juliflora* was highly differentiated from the rest of the (diploid) species within the genus. The lack of genetic differentiation between most diploid species in their native ranges supports the notion that hybridization between allopatric species may occur frequently when they are co-introduced into non-native areas. For regulatory purposes, we propose to treat diploid *Prosopis* taxa from the Americas as a single taxonomic unit in non-native ranges.

## Teaser

Uncertain taxonomy can complicate the management of invasive species. Using population genetic data Castillo *et al.* aimed to clarify the taxonomic placement of selected invasive *Prosopis* populations. They found tetraploid *P. juliflora* to be highly differentiated from all other diploid species of the genus. Diploid species, on the other hand, had low genetic differentiation, supporting anecdotal evidence that suggests these species often form hybrid swarms in their non-native ranges. For regulatory purposes all invasive diploid *Prosopis* taxa should be treated as a single taxonomic unit.

## Introduction

Biological invasions are a major threat to biodiversity, ecosystem services and human well-being (Pimentel *et al.* 2005; van Wilgen *et al.* 2011; Shackleton *et al.* 2014). With globalization, the number of species being translocated, intentionally or accidently, is ever increasing as part of socio-economic development (Seebens *et al.* 2020).

Sound taxonomic knowledge of invasive populations is crucial to detect new invasions, to determine the potential sources and pathways of introduction(s), to prevent or reduce the arrival of new invaders, to accurately model potential ecological niches and to implement management options such as biological control (Le Roux and Wieczorek 2009; Ensing *et al.* 2013). However, the taxonomy of many alien taxa remains problematic due to unresolved phylogenetic relationships, uncertain native-range geographic distributions and interspecific hybridization, among other factors (Pyšek *et al.* 2013). For example, invasive *Heracleum* species belong to a taxonomically complex group, making identification of several invasive taxa difficult (Jahodová *et al.* 2007). In the USA, large areas of riparian and wetland habitats have been invaded by Eurasian saltcedar (*Tamarix*) species. Gaskin and Schaal (2002) found hybridization among *Tamarix* species to be widespread in the invaded range, while levels of hybridization in the native range appear to be low. Under such complex scenarios, complementing ecomorphological approaches (i.e. morphological data and environmental requirements) with genetic information may be critical to delimit species boundaries (Le Roux and Wieczorek 2009).

Comparative ecological and genetic studies between conspecific invasive alien species from different parts of the world provide opportunities to clarify genetic relationships and provide insights into taxonomy and invasion history, i.e. knowing which taxa are invasive and where (Gaskin and Schaal 2002; Gallego-Tévar *et al.* 2019). Research examining genetic diversity and differentiation within and among invasive populations as well as between invasive and native populations, is commonly conducted to unravel introduction histories (Lavergne and Molofsky 2007; Le Roux *et al.* 2010; Hirsch *et al.* 2019), dispersal routes within non-native areas (Lachmuth *et al.* 2010) and the role that genetic constraints play in invasive performance (Dlugosch and Parker 2008). Historical range expansions and past demographic processes may also affect levels of genetic diversity present in invasive populations (Taylor and Keller 2007; Le Roux *et al.* 2011). Lastly, intra- or interspecific hybridization following introduction may replenish species genetic diversity (Ellstrand and Schierenbeck 2000), mask deleterious alleles and cause fixed heterosis (te Beest *et al.* 2012).

The genus *Prosopis* (Leguminosae), commonly known as mesquite, includes some of the world’s worst woody invasive species (Shackleton *et al.* 2014). The taxonomy of *Prosopis* species is problematic because diagnostic morphological traits are often lacking and because the native distributions of many species remain contentious (Pasiecznik *et al.* 2001). Following Burkart (1976), the genus comprises 44 species from the Americas, South West Asia and North Africa, which are mostly found in arid and semiarid regions. *Prosopis* species have been grouped into five sections, from these, the section Algarobia is divided into six series based on leaf morphological traits (Burkart 1976). The validity of these series has been questioned due to taxonomic uncertainty, interspecific hybridization and a probable polyphyletic origin (Bessega *et al.* 2006; Burghardt and Espert 2007; Sherry *et al.* 2011).

*Prosopis* species have been intentionally moved around the globe for many reasons, including for soil stabilization and to provide fuel and livestock fodder. These movements have been characterized by multiple introductions, often of multiple species from various sources, to different localities (Pasiecznik *et al.* 2001). Alien *Prosopis* species are now present in 103 countries and are considered invasive in 49 of these (Shackleton *et al.* 2014). Given the problematic taxonomy of *Prosopis* species many studies simply refer to the taxon as *Prosopis* in their non-native ranges. Taxonomic uncertainty is further exacerbated due to frequent hybridization between different species. For example, in South Africa, numerous species were introduced and became invasive, including *Prosopis glandulosa*, *Prosopis velutina* and *Prosopis laevigata* (Poynton 2009). Here, DNA sequencing data showed that extensive hybridization is occurring and were unable to identify ‘pure’ parental species (Mazibuko 2012). In Australia, introduced populations have been morphologically identified as Prosopis juliflora, *P. glandulosa*, Prosopis pallida, *P. velutina*, and their hybrids (van Klinken and Campbell 2001), with the most severe infestation being represented by a hybrid swarm between *P. pallida* × *P. velutina* × *P. glandulosa* var. *glandulosa* (van Klinken 2012). In Hawaii, morphological hybrids between *P. juliflora* and the invasive *P. pallida* seem to be present in several locations (Gallaher and Merlin 2010).

In Eastern Africa, various *Prosopis* species were introduced and some became invasive. Importantly, there is no credible information available on the origin(s) of *Prosopis* individuals, their introduction histories or their taxonomic classification in this region (Choge *et al.* 2011). In Kenya, *P. juliflora* and *P. pallida* were first introduced in 1973 to Mombasa (Johansson 1990) with later introductions of various *Prosopis* species during the 1970s and 1980s to different parts of Kenya, including Baringo County, Tana River and Taveta (Johansson 1990; Otsamo and Maua 1993; Choge *et al.* 2002; Little 2019). The aggressive spread of *P. juliflora* has been documented (Choge *et al.* 2002; Mbaabu *et al.* 2019; M. L. Castillo *et al.*, unpubl. data), while *P. pallida* has seemingly not become invasive (M. L. Castillo *et al.*, unpubl. data). The overlapping morphological traits and native-range distributions of these two species (Burkart 1976; Díaz Celis 1995) have often led to misidentifications in both native and non-native areas, with some suggesting that they should be treated as a species complex (Pasiecznik *et al.* 2001). Intermediate morphotypes between *P. juliflora* and *P. pallida* have been observed in Kenya, i.e. putative hybrids (W. Okellu, CABI, unpubl. data). In Ethiopia and Tanzania, morphological identification of invasive trees remains unclear and studies only refer to the taxon as *Prosopis* or *P. juliflora* (Wakie *et al.* 2014; Kilawe *et al.* 2017; Shiferaw *et al.* 2019). Hybrids between *P. juliflora* and *P. pallida* are assumed to be absent or rare (Wakie *et al.* 2014; Kilawe *et al.* 2017; Shiferaw *et al.* 2019). In Ethiopia, *Prosopis* was first introduced in the early 1980s into the Afar Region, with additional introductions between the 1980s and 1990s (Admasu 2008; Kebede and Coppock 2015). *Prosopis* is now considered one of the country’s worst invasives. In Tanzania, *Prosopis* was thought to have been first introduced in 1953 to Mombo Arboretum and Tanga region (J. R. Mbwambo, Tanzania Forestry Research Institute, pers. comm.), with later introductions between 1988 and 1995 (Kilawe *et al.* 2017). *Prosopis* is considered to be at an early stage of invasion in Tanzania. 

Studies at large biogeographic scales, including both native and non-native ranges, may provide valuable information about the genetic diversity and differentiation of invasive *Prosopis* species, the occurrence of hybridization, and may help clarify taxonomic uncertainties. In this study, we assessed the genetic diversity, differentiation and structure, and evaluated the occurrence of interspecific hybridization in native and non-native populations of several *Prosopis* species, with a special focus on non-native populations of *P. juliflora* and *P. pallida* in Eastern Africa (Ethiopia, Kenya and Tanzania). For the latter region, we also wanted to indirectly infer the introduction histories of both *Prosopis* species by comparing levels of genetic diversity and differentiation between native and Eastern African populations.

## Materials and Methods

### Sampling and DNA extraction

**Figure 1.**
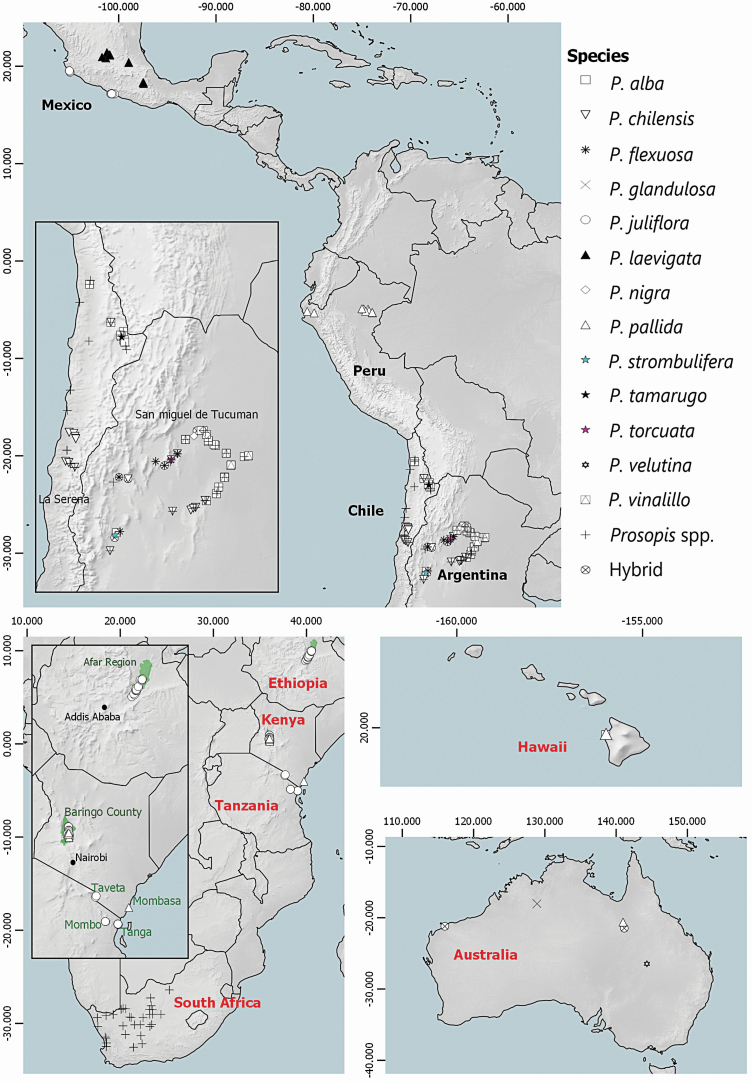
Sampling sites of various *Prosopis* taxa and putative hybrids from native (black labels) and non-native areas (red labels). Inset maps indicate sampling sites in Argentina, Chile, Ethiopia, Kenya and Tanzania.

Leaf material of different *Prosopis* species was collected from various native and non-native areas worldwide in 2016 (Table 1; Fig. 1). For this, sampling in the native areas was done within the two known centres of diversification of the genus, the Argentine–Paraguayan–Chilean region and the Texan–Mexican region. We aimed to include a high number of species, rather than sampling comprehensively across the distributions of only a few species. From the native range, we sampled individuals of *P. juliflora* and *P. laevigata* from Mexico; *P. pallida* from Peru; *Prosopis alba*, *Prosopis chilensis*, *Prosopis flexuosa*, *Prosopis nigra*, *Prosopis strombulifera*, *Prosopis torcuata* and *Prosopis vinalillo* and putative hybrids from Argentina, as well as *Prosopis tamarugo*, *P. chilensis* and *P. alba* from Chile. A total of 67 sampled individuals from native areas in Argentina and Chile could not be morphologically identified to species level (hereafter referred to only as *Prosopis* spp.). The native ranges of sampled species are provided in **Supporting Information—Appendix S1**.

**Table 1.** *Prosopis* species included in the study from various native, introduced and invasive populations. For each species, the number of individuals (*N*) sampled in each country, its status in each country (native, introduced or invasive) and the section/series where it belongs to, are shown. The series Chilenses, Pallidae and Ruscifoliae are part of the section Algarobia.

Species	Country	*N*	Status	Section/series
*P. alba*	Argentina	29	Native	Series Chilenses
	Chile	11	Native	
*P. chilensis*	Argentina	19	Native	Series Chilenses
	Chile	25	Native	
*P. flexuosa*	Argentina	9	Native	Series Chilenses
*P. glandulosa*	Australia	2	Invasive	Series Chilenses
*P. juliflora*	Mexico	20	Native	Series Chilenses
	Ethiopia	202	Invasive	
	Kenya	470	Invasive	
	Tanzania	50	Invasive	
*P. laevigata*	Mexico	25	Native	Series Chilenses
*P. nigra*	Argentina	6	Native	Series Chilenses
*P. pallida*	Peru	14	Native	Series Pallidae
	Australia	2	Invasive	
	Hawaii	15	Invasive	
	Kenya	57	Introduced	
*P. strombulifera*	Argentina	1	Native	Section Strombocarpa
*P. tamarugo*	Chile	1	Native	Section Strombocarpa
*P. torquata*	Argentina	1	Native	Section Strombocarpa
*P. velutina*	Australia	1	Invasive	Series Chilenses
*P. vinalillo*	Argentina	7	Native	Series Ruscifoliae
*P. alba* × *P. chilensis*	Argentina	2	Native	
*P. alba* × *P. nigra*	Argentina	3	Native	
*P. alba* × *P. rustifolia*	Argentina	1	Native	
*P. alba* × *P. vinalillo*	Argentina	1	Native	
*P. chilensis* × *P. flexuosa*	Argentina	4	Native	
Hybrids	Australia	4	Invasive	
*Prosopis* spp.	South Africa	58	Invasive	
*Prosopis* spp.	Argentina	35	Native	
*Prosopis* spp.	Chile	32	Native	
	Total	1107		

From non-native areas, we sampled individuals of P. glandulosa, *P. pallida*, *P. velutina* and putative hybrids from Australia, *P. pallida* from Hawaii and various *Prosopis* species and putative hybrids from South Africa that could not be identified to species level (hereafter referred to only as *Prosopis* spp.). From Eastern Africa, we included areas where *P. juliflora* and *P. pallida* individuals were first introduced; we sampled *P. juliflora* from the Afar Region, Ethiopia, *P. juliflora* and *P. pallida* from Baringo County, Mombasa and Taveta, Kenya, and *P. juliflora* from Mombo and Tanga arboreta, Tanzania. All sampled species belong to the sections Algarobia and Strombocarpa, and from the section Algarobia, the species belong to the series Chilenses, Pallidae and Ruscifoliae (Table 1). One to 35 sampling sites were included per country for each species and 1–474 adult trees were sampled per species per country (Table 1; Fig. 1; *n*_total_ = 1107 individuals). This uneven sampling reflects the availability of individuals at each location, i.e. areas where only one tree was located versus areas with dense invasive populations. We sampled trees that were separated by at least 30 m to avoid collecting genetically related material (Vilardi *et al*. 1988). In the case of Kenya, Ethiopia and Tanzania, some sampled trees were separated by less than 30 m. Throughout this manuscript we use the term ‘population’ to refer to a group of individuals of the same species that were sampled in a specific native or non-native area in each country. Leaf material was air-dried and stored on silica gel until further use for DNA extraction. Because *P. juliflora* is the only polyploid member of the genus (2*n* = 4*x*), we performed flow cytometry analysis on a subset of *P. juliflora* individuals (*n* = 75). Further details on DNA extraction, morphological classification of individuals and flow cytometry analyses are provided in **Supporting Information—Appendix S1**.

### Nuclear and chloroplast DNA sequencing

To assess evolutionary history (i.e. phylogeny) of our study species, we optimized and sequenced the nuclear external transcribed spacer region (*ETS*) and two chloroplast intergenic spacers (*rpl32-trnL* and *psbA*-*trnH*) for 12 *Prosopis* individuals initially to check for genetic variability at these gene regions prior to sequencing a more representative sample of individuals. *ETS* and *rpl32-trnL* have previously been successfully employed at the intraspecific level for Leguminosae species (e.g. Australian *Acacia* species; Le Roux *et al.* 2011) and the *psbA*-*trnH* marker is generally highly variable across angiosperms (Shaw *et al.* 2007). Details of the *Prosopis* individuals selected for initial screening, primers used for PCR amplification are provided in **Supporting Information—Appendix S1**. DNA sequence data gene were aligned and edited for each gene region separately using BioEdit version 7.0.5.3 (Hall 1999).

### Microsatellite genotyping

We selected 11 nuclear microsatellite markers considering their levels of polymorphism across different *Prosopis* species, functional annotations in some instances and similar annealing temperatures **[see**  **Supporting Information—Table S1****]**. Details of marker amplification and genotyping are provided in **Supporting Information—Table S2** and **Appendix S1**. From these markers, the following four loci were excluded from subsequent analyses: I-P00930c as it was monomorphic; I-P07653, GL23 and Prb8 as these showed extensive patterns of non-specific binding in numerous samples. Samples that failed to amplify at more than five loci were removed from all subsequent analyses (including the single *P. tamarugo* individual), leaving a total of 1072 individuals. Individuals not identified as *P. juliflora* and that had more than two alleles at least one locus were excluded from subsequent analyses because their ploidy could not be reliably determined (*n* = 14; **see**  **Supporting Information—Table S3**).

### Genetic diversity and differentiation between native and non-native populations

Departures from Hardy–Weinberg equilibrium (HWE) were tested for all loci for all diploid species (i.e. excluding *P. juliflora*) using the R packages *adegenet* version 2.0.1 (Jombart 2008) and *pegas* version 0.11 (Paradis 2010) and significance was tested using a permutation test (10 000 permutations). We calculated various statistics of genetic diversity separately for all native and non-native populations of various *Prosopis* species, putative hybrids from Australia and *Prosopis* spp. individuals from South Africa. For these analyses, all putative hybrids from Argentina were analyzed as a single taxon, and species for which we were only able to collect one or two individuals at a particular location were not included in the analysis. A total of 18 populations were included in the analyses. In addition, to evaluate whether genetic diversity differs between native and non-native populations of *Prosopis*, we grouped all native-range individuals of all *Prosopis* species, putative hybrids and *Prosopis* spp. individuals, referred to hereafter as ‘Native *Prosopis*’. Separately, we grouped all non-native-range individuals (i.e. introduced and invasive) of all *Prosopis* species, putative hybrids and *Prosopis* spp. individuals, referred to hereafter as ‘Non-native *Prosopis*’. Then, we estimated genetic diversity indexes separately for each group and compared them. To assess levels of genetic diversity, numbers of alleles per locus, observed heterozygosity (*H*_O_), expected heterozygosity (*H*_E_) and inbreeding coefficients (*F*_IS_) were estimated using the SPAGeDi version 1.5 software for polyploid *P. julifora* (Hardy and Vekemans 2002). For all diploid individuals, *H*_O_ and *H*_E_ were estimated with the software GenoDive version 3.0 (Meirmans 2020), and numbers of alleles per locus and *F*_IS_ values were estimated with the *diveRsity* R package version 1.9.90 (Keenan *et al.* 2013). Since there is a positive correlation between population size and *H*_E_ (Nybom 2004), GenoDive and SPAGeDi analyses included corrections for sample sizes for *H*_E_ calculations. Lastly, allelic richness (*A*_R_) and the number of private alleles were calculated with the software ADZE version 1.0 (Szpiech *et al.* 2008). ADZE uses a rarefaction approach to calculate sample size-corrected estimates for these metrics. The number of individuals included in these analyses per population is reported in Table 2.

**Table 2.** Population genetic diversity indices for native, and non-native (introduced and invasive) populations of various *Prosopis* species, putative hybrids and *Prosopis* spp. individuals from South Africa. Native *Prosopis* and non-native *Prosopis* groups (i.e. all native and non-native *Prosopis* individuals, respectively) were analysed as well. Statistics were calculated as mean values of each index over the seven loci analysed. *N* = number of samples; *H*_E_ = expected heterozygosity expected; *H*_O_ = observed heterozygosity observed; *F*_IS_ = inbreeding coefficient.

Species	Country	Category	*N*	*H*_E_	*H*_O_	*F*_IS_
*P. alba*	Argentina	Native	28	0.71	0.65	0.05
*P. alba*	Chile	Native	11	0.68	0.49	0.22
*P. alba*		**All**	**39**	**0.70**	**0.57**	**0.13**
*P. chilensis*	Argentina	Native	9	0.69	0.57	0.09
*P. chilensis*	Chile	Native	24	0.66	0.55	0.15
*P. chilensis*		**All**	**43**	**0.68**	**0.56**	**0.16**
*P. flexuosa*	Argentina	Native	8	0.70	0.60	0.02
*P. juliflora*	Mexico	Native	20	0.31	0.44	-0.20
*P. juliflora*	Ethiopia	Invasive	200	0.35	0.42	0.08
*P. juliflora*	Kenya	Invasive	457	0.42	0.46	0.18
*P. juliflora*	Tanzania	Invasive	46	0.28	0.29	0.10
*P. juliflora*		**All**	**723**	**0.41**	**0.44**	**0.19**
*P. laevigata*	Mexico	Native	24	0.48	0.47	0.33
*P. nigra*	Argentina	Native	6	0.57	0.47	0.01
*P. pallida*	Peru	Native	12	0.42	0.30	0.30
*P. pallida*	Hawaii	Invasive	14	0.29	0.20	0.24
*P. pallida*	Kenya	Introduced	57	0.39	0.28	0.21
*P. pallida*		**All**	**83**	**0.37**	**0.26**	**0.22**
*P. vinalillo*	Argentina	Native	7	0.68	0.58	0.09
Hybrids	Argentina	Native	10	0.71	0.68	-0.08
Hybrids	Australia	Invasive	3	0.66	0.33	0.41
*Prosopis* spp.	South Africa	Invasive	48	0.69	0.58	0.14
Native *Prosopis*			**229**	**0.62**	**0.49**	**0.26**
Non-native *Prosopis*			**829**	**0.45**	**0.36**	**0.32**

We also estimated genetic differentiation between native and non-native populations of various *Prosopis* species, putative hybrids from Argentina and Australia, and *Prosopis* spp. individuals from South Africa as described above (i.e. 18 populations and the same number of individuals per population as detailed in Table 2). For *P. juliflora*, a matrix of pairwise genetic distances (*F*_ST_) was calculated using the R package *PolySat* with 95 % confidence intervals calculated via bootstrapping across loci. For diploid individuals, pairwise *F*_ST_ values were calculated following Weir (1996). For this, the FreeNA software (Chapuis and Estoup 2007) was used to calculate corrected and uncorrected *F*_ST_ estimates since it applies an ‘excluding null alleles’ (ENA) correction to account for the presence of null alleles. The 95 % confidence intervals for *F*_ST_ values were obtained by 10 000 simulations. *F*_ST_ estimates depend on within-population genetic diversity and therefore, on sample sizes (Meirmans and Hedrick 2011). Therefore, we also calculated pairwise *G″*_ST_ estimates, which includes a correction for sampling bias, using the GenoDive software (Meirmans and Hedrick 2011). In addition, a hierarchical analysis of molecular variance (AMOVA) was performed including native and non-native *P. juliflora* and *P. pallida* populations of Ethiopia, Kenya and Tanzania and using the *pegas* R package (Paradis 2010). For *P. juliflora*, a matrix of pairwise distances between individuals was generated using Bruvo distances (Bruvo *et al.* 2004), while for *P. pallida*, Euclidian distance based on the allele frequencies was used to generate pairwise distances between individuals.

### Genetic structure and hybridization

To identify the number of genetic clusters present in the overall data set, Bayesian assignment tests were used as implemented in the software STRUCTURE version 2.3.4 (Pritchard *et al.* 2000). A hierarchical clustering approach (Le Roux *et al.* 2010) was applied including native and non-native populations of all investigated *Prosopis* species, putative hybrids and *Prosopis* spp. individuals. Details of model parameters and settings are provided in **Supporting Information—Appendix S1**.

Principal component analyses (PCAs) were also performed. A first PCA included native and non-native populations of all *Prosopis* species, their putative hybrids and *Prosopis* spp. individuals. We used the *PolySat* R package (Clark and Jasieniuk 2011) to generate a matrix of pairwise distances between individuals using Bruvo distances since this method can incorporate distances between microsatellite alleles without information on allele copy number (Bruvo *et al.* 2004). In a second ‘*diploid-only*’ PCA (i.e. excluding *P. juliflora* individuals), we generated a matrix of Euclidian distances between individuals considering allele frequencies.

We tested the morphological assignment of diploid individuals to pure species and putative hybrids using microsatellite data and the NewHybrids version 1.1beta software (Anderson and Thompson 2002). This software identifies six genotype classes (i.e. pure species 1, pure species 2, F1 hybrids, F2 hybrids, species 1 backcrosses and species 2 backcrosses) without information on the allele frequency of the parental species. The program provides probabilities of an individual belonging to any of the genotype classes and therefore how well our *a priori* morphological assignment of individuals aligned with the genetic data. An analysis was done between all possible pairs of species from Argentina: *P. alba*, *P. chilensis*, *P. flexuosa*, *P. nigra*, *P. vinalillo* and their putative hybrids. In the case of Chile, the analysis was done between *P. alba* and *P. chilensis* individuals. A last analysis was done between Peruvian *P. pallida* individuals and Hawaiian and Kenyan *P. pallida* individuals. A burn-in period of 30 000 generations and 50 000 MCMC iterations was used. We used ‘Jeffrey’s like priors’ and a posterior probability of 0.8 was used to assign individuals to the six genotype classes. Individuals that could not be assigned to genotype classes were considered of ‘mixed’ ancestry.

## Results

### Nuclear and chloroplast DNA sequencing

DNA sequencing data for the *ETS*, *psbA*-*trnH* and *rpl32-trnL* gene regions indicate extremely low sequence variability across our initial subset of *Prosopis* species from native and non-native populations. The exception was *P. tamarugo*, where we found 15 substitutions for the *psbA*-*trnH* region, 17 substitutions for the *rpl32-trnL* region and ~240 substitutions for the *ETS* region. When excluding *P. tamarugo*, we found only one substitution in the *psbA*-*trnH* region between *P. pallida* from Peru and all other species, and one substitution in the *rpl32-trnL* region between *P. nigra* and *P. flexuosa* and all other species. For the *ETS* region, we found only four substitutions between *P. juliflora* from Kenya and all other studied species. *Prosopis* spp. from South Africa differed by three substitutions with all other species, while *P. glandulosa* and the putative hybrids from Australia, and *P. pallida* from Peru had one substitution each when compared with the rest of the studied species. Given this low differentiation between species in the Algarobia section we did not sequence additional individuals.

### Genetic diversity and differentiation between native and non-native populations

For the microsatellite data and for diploid *Prosopis* species, 21 loci for each species by country combination (27.3 %) did not meet HWE expectations. All seven loci were polymorphic in the overall data set. Four markers were not polymorphic for some native and non-native populations of *Prosopis* species and putative hybrids from Australia **[see**  **Supporting Information—Table S4****]**. The average number of alleles per locus was 17.4 (range 6–33 alleles).

**Figure 2.**
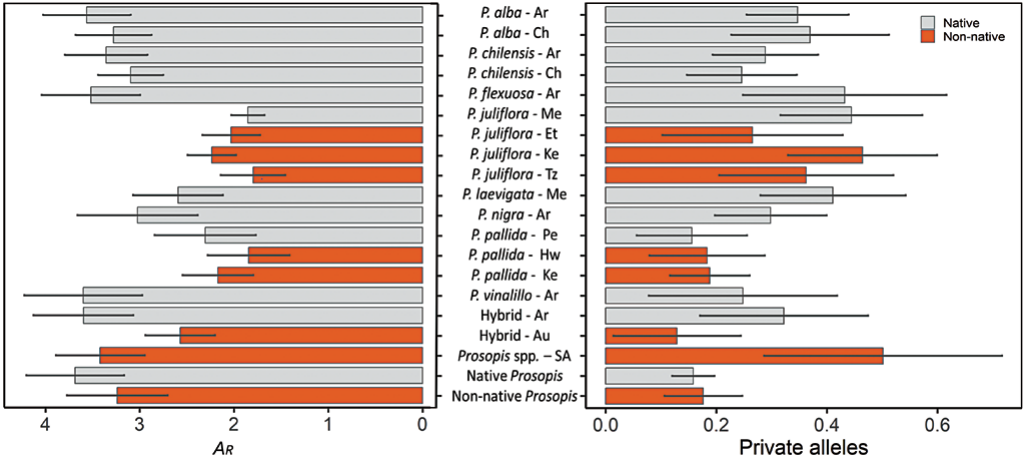
Allelic richness (*A*_R_) and number of private alleles_._ (±1 SE) for native and non-native populations of various *Prosopis* species, putative hybrids and *Prosopis* spp. individuals from South Africa. Native *Prosopis* and non-native *Prosopis* groups (i.e. all native and non-native *Prosopis* individuals, respectively) were analyzed as well. Country codes are: Argentina (Ar), Australia (Au), Chile (Ch), Ethiopia (Et), Hawaii (Hw), Kenya (Ke), Mexico (Me), South Africa (SA) and Tanzania (Tz).

Overall, native populations of pure *Prosopis* species, putative hybrids from Argentina and *Prosopis* spp. individuals from South Africa had higher numbers of alleles per locus, levels of *A*_R_, *H*_E_ and *H*_O_ than native and non-native populations of *P. juliflora* and *P. pallida* and Australian putative hybrids (Table 2; Fig. 2). Inbreeding coefficients (*F*_IS_) were high in most native and non-native populations of *Prosopis* species. The number of private alleles was similar among native and non-native populations of *Prosopis* species and putative hybrids from Argentina (Table 2).

In the case of *P. juliflora*, native Mexican populations had lower *H*_E_ compared to invasive population from Kenyan, but higher *H*_E_ and *H*_O_ than invasive Tanzanian populations. Native and non-native populations of *P. pallida* had similar levels of *A*_R_. In the case of *P. pallida*, native Peruvian populations had similar *H*_E_ and *H*_O_ than introduced Kenyan populations. Invasive Hawaiian populations had lower *H*_E_ and *H*_O_ compared to native and other non-native populations of the species. Lastly, native *Prosopis* populations (i.e. Native *Prosopis*) had higher levels of *A*_R_, *H*_E_ and *H*_O_ than the non-native populations (i.e. non-native *Prosopis*), but lower levels of *F*_IS_. The number of private alleles was similar between these groups (Fig. 2).

When estimating genetic differentiation, similar results were obtained with uncorrected and ENA-corrected pairwise *F*_ST_ estimates (Kruskal–Wallis chi-square = 0.03, *P* = 0.85); therefore, uncorrected pairwise *F*_ST_ values with 95 % confidence intervals are presented **[see**  **Supporting Information—Tables S5**  **and**  **S6****]**. Overall, we found low genetic differentiation between some *Prosopis* species and hybrids in spite of their allopatric distributions. Levels of differentiation based on pairwise *G″*_ST_ estimates were similarly low **[see**  **Supporting Information—Table S7****]**. For example, genetic distances (i.e. pairwise *F*_ST_ and *G″*_ST_ values) between some sympatric *Prosopis* species from Chile and Argentina were similar to those between these species and *P. laevigata* from Mexico, *Prosopis* spp. individuals from South Africa and putative Australian hybrids.

**Figure 3.**
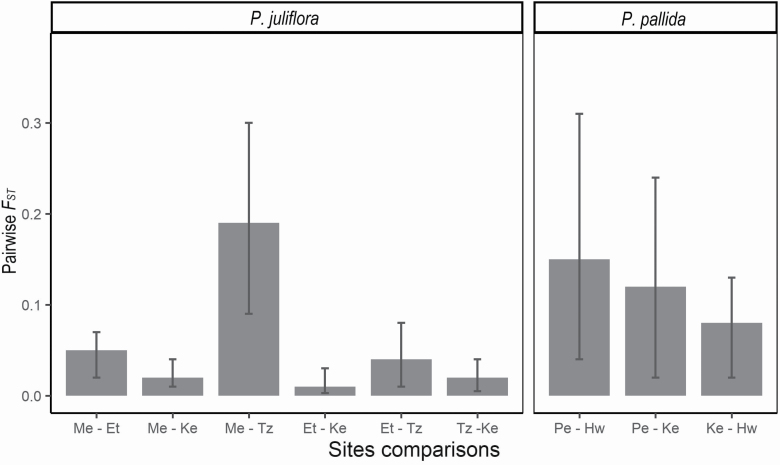
Pairwise *F*_ST_ (± 95 % confidence interval) between native Mexican (Me) and invasive populations of *P. juliflora* in Ethiopia (Et), Kenya (Ke) and Tanzania (Tz); between invasive populations of *P. juliflora*; between native populations from Peru (Pe) and invasive populations from Hawaii (Hw) and introduced populations from Kenya (Ke) and between Ke and Hw populations of *P. pallida*.

Regarding *P. juliflora*, *F*_ST_-based genetic differentiation between invasive Tanzanian and native Mexican populations was higher than between the latter and invasive populations from Kenya and Ethiopia, and was also higher than the differentiation between invasive Kenyan and Ethiopian populations (Fig. 3). These results were also supported by pairwise *G″*_ST_ estimates (**see**  **Supporting Information—Table S7**). The hierarchical AMOVA indicated considerable, but not significant, genetic variation between native and non-native *P. juliflora* populations (71.36 %), while significant, and similar, genetic variation was found among invasive populations (12.32 %) and within invasive populations (16.32%; Table 3). In the case of *P. pallida*, levels of genetic differentiation (based on *F*_ST_ and *G″*_ST_) were similar between native Peruvian populations and non-native (both introduced and invasive) populations from Hawaii and Kenya (Fig. 3; **see**  **Supporting Information—Table S7**). There was also some, but not significant, genetic variation between native and non-native populations of *P. pallida* (37.29 %), while the genetic variation between native, introduced and invasive populations (28.66 %) was significant and slightly lower than the variation within populations (34.05 %; Table 3).

**Table 3.** Hierarchical AMOVA partitioning of genetic variation for various native, introduced and invasive populations of *P. juliflora* and *P. pallida*. * Significant fixation indices, tested using 10 000 random permutations. d.f. = degrees of freedom.

Source of variation	d.f.	Sum of squares	Variance	Percent variation (%)	Fixation index
*P. juliflora*					
Native versus non-native populations	1	1.28	170.02	71.36	0.45
Among native and invasive populations	2	1.58	29.36	12.32	0.13*
Within populations	710	21.87	38.88	16.32	0.52
*P. pallida*					
Native versus non-native populations	1	37.71	22.48	37.29	0.18
Among native, introduced and invasive populations	1	13.53	17.28	28.66	0.23*
Within populations	80	432.18	20.53	34.05	0.06

### Genetic structure and hybridization

**Figure 4.**
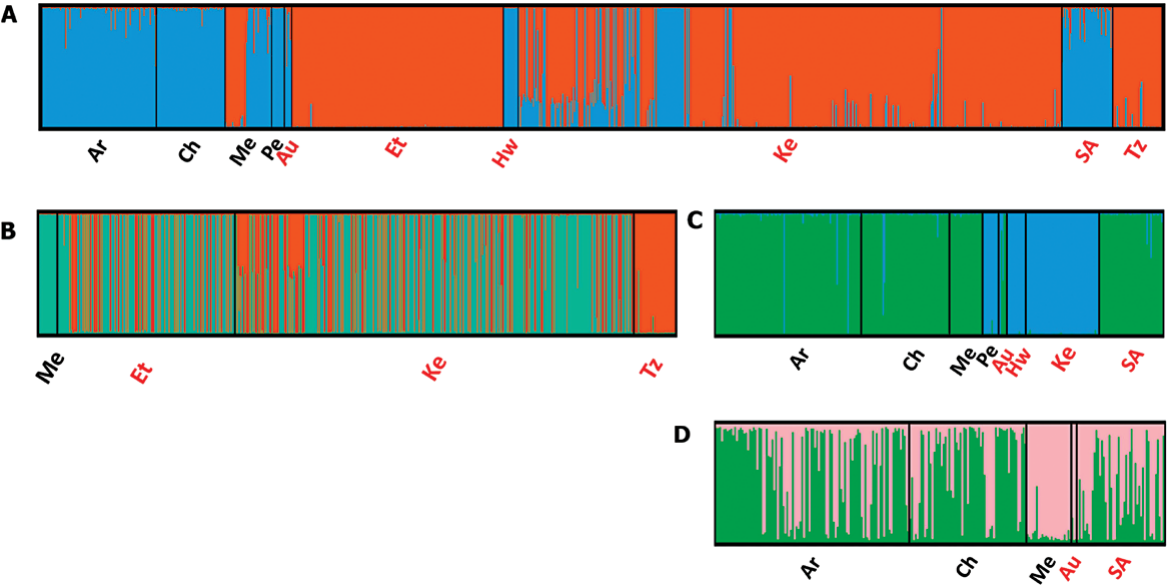
Hierarchical Bayesian clustering analyses of individuals of native (black labels) and non-native (red labels) populations of various *Prosopis* species and putative hybrids: Argentina (Ar) = P. alba, *P. chilensis*, *P. flexuosa*, *P. strombulifera*, *P. nigra*, *P. torcuata* and *P. vinalillo*, putative hybrids and *Prosopis* spp. individuals; Chile (Ch) = *P. alba*, *P. chilensis*, and *Prosopis* spp. individuals; Mexico (Me) = *P. juliflora* and *P. laevigata*; Peru (Pe) = *P. pallida*; Australia (Au) = *P. glandulosa*, *P. pallida*, *P. velutina* and putative hybrids; Ethiopia (Et) = *P. juliflora*; Hawaii (Hw) = *P. pallida*; Kenya (Ke) = *P. juliflora* and *P. pallida*; South Africa (SA) *= Prosopis* spp. individuals; Tanzania (Tz) = *P. juliflora*. Individuals were genotyped using seven nuclear microsatellite loci and clustered at three levels. (A) Level 1: ‘*P. juliflora*’ cluster in orange and ‘other *Prosopis* species’ cluster in blue; (B) Level 2: only *P. juliflora* individuals and (C) Level 2: ‘*P. pallida*’ cluster in blue ‘other *Prosopis* species’ cluster in green; (D) Level 3: individuals of ‘other *Prosopis* species’ cluster from Argentina, Chile, Mexico, Australia and South Africa. Vertical axes represent the assignment (*q*_*ik*_ values) of individual genomes to the inferred number of genetic clusters, in all cases *K* = 2 **[see**  **Supporting Information—Fig. S1****]**.

**Figure 5.**
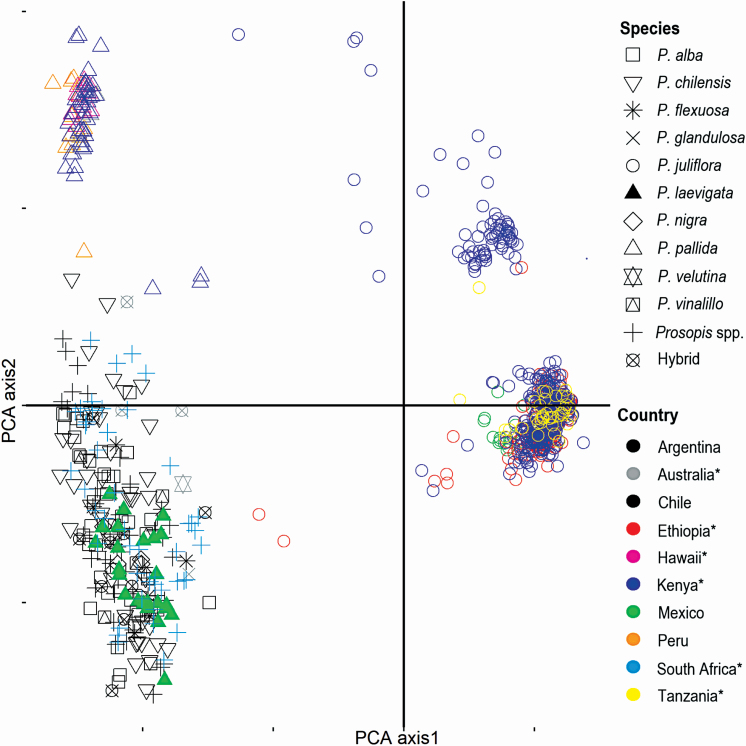
Principal component analysis (PCA) showing genetic structure among native and non-native populations of different *Prosopis* taxa and their putative hybrids. Countries from which non-native populations originated are indicated by asterisks (*). PCA was performed using Bruvo distances calculated in *PolySat* (Bruvo *et al.* 2004). PCA 1 and PCA 2 captured 63.6 % and 11.0 % of the variation, respectively.

Both Bayesian assignment tests and PCAs indicated that overall genetic structure largely reflected ploidal variation, with polyploid *P. juliflora* being highly differentiated from the rest of the diploid *Prosopis* species included here. A second level of hierarchical structure largely reflected series-level relationships, showing genetic differentiation between *P. pallida* and the remaining diploid species, while there was not a clear genetic structure among *Prosopis* species from Argentina, Australia, Chile, Mexico and Prosopis spp. from South Africa. Interestingly, both analyses confirmed the presence of *P. juliflora* in Ethiopia, Kenya and Tanzania, and also identified some admixed individuals, i.e. hybrids, in Kenya. Lastly, STRUCTURE, but not PCA, showed low genetic differentiation between native Mexican *P. juliflora* populations and invasive populations from Ethiopia and Kenya (Figs 4 and 5; **see**  **Supporting Information—Figs S1**  **and**  **S2**).

Assignment tests in NewHybrids were done between pairs of species per site **[see**  **Supporting Information—Fig. S3****]**. These analyses were able to identify only three genotype classes: pure parental species and their hybrids. For most comparisons including species from Argentina and Chile, individuals morphologically identified as one of the two species, aligned with the genetic data and were assigned as pure genotypes of the same species between 57.1–100 % of assignments. In contrast, individuals were also assigned as pure genotypes of the other species (3.7–21.1 % of assignments); or as having mixed ancestry (5.3–42.9 % of assignments). Only when including pairs of the species *P. flexuosa*–*P. nigra* and *P. flexuosa*–*P. vinalillo* from Argentina, were the models unable to assign individuals to any class. Interestingly, for two of the three putative Argentinean hybrids, morphological identification did not align well with the genotype classification. That is, morphological hybrids were genetically mainly classified as being one of the pure parental species (66.7 %). Lastly, an analysis between *P. pallida* individuals from Peru and *P. pallida* individuals from Hawaii and Kenya classified all Peruvian individuals as pure parental genotypes. For *P. pallida* from Hawaii, half of the individuals represented pure genotypes that differ from Peruvian genotypes, few of them were classified as pure *P. pallida* genotypes from Peru (7.14 % of assignments) and the rest were found to have mixed ancestry (42.9 %). In contrast, almost all *P. pallida* individuals from Kenya were classified as pure Peruvian *P. pallida* genotypes (92.0 %) and a few as having mixed ancestry (8.0 %).

## Discussion

While numerous studies have reported on the genetic relationships among *Prosopis* species and population-level genetic variation (e.g. Ramírez *et al.* 1999; Saidman *et al.* 2000; Bessega *et al.* 2006; Catalano *et al.* 2008; Moncada et al. 2019; Aguilar *et al.* 2020), ours is the first to provide genetic insights on the taxonomic uncertainty of non-native *Prosopis* species. We found low genetic differentiation between most diploid *Prosopis* species, suggesting that hybridization between previously allopatric species may occur frequently when they are co-introduced into new ranges. Polyploid individuals were detected in both native and non-native areas, with tetraploid *P. juliflora* being highly differentiated from the rest of the diploid species in the genus. Levels of genetic diversity suggest that invasive populations in Eastern Africa (Kenya and Ethiopia) resulted from multiple introductions of both *P. juliflora* and *P. pallida*. While hybridization is thought to promote invasiveness of *Prosopis* in countries like Australia and South Africa, this seems not be the case in Eastern Africa. Here polyploidy appears to benefit invasion success.

### Uncertain taxonomy of diploid *Prosopis taxa*

The taxonomy of *Prosopis* has been much debated (Saidman and Vilardi 1987; Saidman *et al.* 2000; Pasiecznik *et al.* 2001). Low genetic variability among diploid taxa has been postulated to blur species boundaries, with some authors considering Algarobia species to constitute a so-called ‘syngameon’, i.e. a hybrid swarm (Palacios and Bravo 1981). Pre-zygotic reproductive barriers (e.g. differences in phenology or the use of different pollinators) are thought to be weak in *Prosopis*, while post-zygotic reproductive barriers (i.e. pollen inviability) may be more important (Palacios and Bravo 1981; Naranjo *et al.* 1984). Our DNA sequencing data indicated that many Algarobia species shared almost 100 % genetic similarity. These results suggest a recent radiation of these species and possibly incomplete reproductive isolation between them (also see Catalano *et al.* 2008). This may lead to frequent hybridization and introgression between species in this section (Hunziker *et al.* 1986), especially when they are co-introduced into new ranges (e.g. van Klinken *et al.* 2006; Mazibuko 2012). Only *P. juliflora* was found to be highly differentiated from the rest of Algarobia species included in our analyses.

We found genetic diversity in *Prosopis* populations to be high and similar among species and native and non-native regions, with the exception of *P. pallida* and *P. juliflora* (also see Juárez-Muñoz *et al.* 2006; Sherry *et al.* 2011). We also identified *Prosopis* individuals that had more than two alleles at some loci in both native and non-native areas. These individuals were not initially classified as tetraploid *P. julifora* based on morphology, but rather as hybrids from Australia, *P. flexuosa* from Argentina, *P. laevigata* from Mexico and individuals from South Africa that could not be identified to species level but are presumed to be hybrids. While polyploidy has been reported in *Prosopis* (Burkart 1976; Hunziker *et al.* 1986; Fontana *et al.* 2018), Trenchard *et al.* (2008) proposed that *P. juliflora* is the only polyploid species in the genus. Ploidal variation is an important mechanism that underlies reproductive isolation, and thus could be promoting genetic differentiation between *P. juliflora* and its congeners.

Taxonomic uncertainty in *Prosopis* was further illustrated by our genetic analysis of hybridization. We found consistent disagreement between taxonomic classification of *Prosopis* species based on morphological versus on genetic data. One possible explanation for this is that hybridization, followed by extensive backcrossing, can lead to individuals expressing the morphological traits of one parental species while retaining genetic information of the other (e.g. see Gaskin and Kazmer 2009; Boswell *et al.* 2016). We also used leaf morphological traits (see Burkart 1976) to classify our species; however, these can be highly plastic (Bessega *et al.* 2006, 2009; Verga *et al.* 2009). Future research should focus on identifying diagnostic traits, and their heritability, for different *Prosopis* taxa.

Our study also provides clarity on the identity of *Prosopis* species in Eastern Africa. Firstly, our genetic results confirmed the presence of *P. juliflora* in Ethiopia, Kenya and Tanzania. Our results using native and non-native genetic material, together with previous work from the native range (Catalano *et al.* 2008; Palacios *et al.* 2012), also indicate important genetic differences between *P. pallida* and *P. juliflora*, confirming that they are indeed distinct taxa. Secondly, we identified a few instances of hybridization between these two species. These hybrids are likely to be triploid and sterile and therefore unlikely to increase invasiveness.

### *Prosopis* invasion in Eastern Africa

Our study provides the first genetic analysis of the origins of *P. juliflora* and *P. pallida* in Eastern Africa. Importantly, in Kenya, Ethiopia and Tanzania, we included comprehensive sampling from areas where *P. juliflora* and *P. pallida* individuals were first introduced, and became invasive, in the case of *P. juliflora*. We found that genetic material of *P. juliflora* appears to be similar for most Kenyan and Ethiopian individuals, and closely related to native Mexican ones. These results suggest that invasive Kenyan and Ethiopian genotypes could have a similar Mexican origin. In the case of *P. pallida* in Kenya, individuals were genetically similar to Peruvian individuals, indicating a South American origin. Additionally, similar levels of genetic diversity were observed between Mexican *P. juliflora* and invasive Ethiopian populations and Peruvian *P. pallida* and introduced Kenyan individuals. In contrast, invasive Kenyan individuals of *P. juliflora* had higher heterozygosity than native individuals from Mexico. It is surprising that is not the case in Ethiopia given similar introduction histories shared by these two countries. Similar or higher levels of genetic diversity in native and non-native populations may be indicative of multiple introductions or it may simply reflect a unique introduction from a source generated by admixture of multiple populations (Le Roux *et al.* 2011). Higher levels of genetic diversity than native individuals can also occur due to cultivation, and can generate genetic novelties (Thompson et al. 2012). Compared to traditional statements that multiple introductions characterize the introduction of *Prosopis* species to non-native areas globally (Pasiecznik *et al.* 2001), our study is the first to provide support to this hypothesis for *P. juliflora* and *P. pallida* in Kenya and Ethiopia.

For Tanzania, the origin and identity of *Prosopis* is more complicated and the available evidence limited. The source(s) and species identity of *Prosopis* individuals originally introduced to two arboreta in the 1960s remains unknown and have been speculated to include *P. juliflora* from other non-native regions like India, Israel and/or South Africa (C. J. Kilawe and J. R. Mbwambo, Tanzania Forestry Research Institute, pers. comm.). However, it is thought that *P. chilensis* and *P. pallida* have also been introduced to Tanzania (C. J. Kilawe, Sokoine University of Agriculture, Tanzania, and J. R. Mbwambo, Tanzania Forestry Research Institute, pers. comm.). Our genetic analyses showed that the trees we collected from two arboreta were *P. juliflora*, similar to *some* genotypes from Ethiopia and Kenya but not closely related to native Mexican ones (Fig. 4B). In other areas of Tanzania, not included in this study, where *Prosopis* is invasive (i.e. Kahe, Mwanga and Simajiro), repeated introductions would have been made from Taita Taveta (C. J. Kilawe, Sokoine University of Agriculture, Tanzania, pers. comm.). It is therefore likely that these invasive populations are also *P. juliflora* since trees collected in Taita Taveta were identified as this species and most individuals were assigned to the same genetic cluster than Tanzanian individuals (results not shown). Therefore, our genetic results showed that, unlike in Kenya and Ethiopia, Tanzanian *P. juliflora* genotypes in arboreta are not closely related to the Mexican individuals, supporting the notion of additional and unknown sources for Tanzanian plantings.

### Hybridization, polyploidy and invasiveness in *Prosopis*

In agreement with previous studies, we identified instances of hybridization between *Prosopis* species in both native (e.g. see Saidman *et al.* 2000) and non-native ranges (e.g. see Zimmermann 1991; van Klinken *et al.* 2006; Mazibuko 2012; Muturi 2012). The success of many plant invasions has been attributed to hybridization (Schierenbeck and Ellstrand 2009; Zalapa *et al.* 2010; Gaskin *et al.* 2012) and this may also be the case for some *Prosopis* invasions such as those in Australia and South Africa (van Klinken *et al.* 2006; Mazibuko 2012), but not in Eastern Africa. In the native range, hybridization between *Prosopis* species seems to be promoted by certain environmental conditions (Vega and Hernández 2005), with hybrids frequently found in disturbed areas (Verga 2005). Considering this, interspecific hybridization between *Prosopis* species in the invaded range may not only be dependent on the genetic relatedness of species, but also on whether certain habitat features facilitate co-occurrence of, and interbreeding between, them. It may also be that only certain *Prosopis* genotypes, or hybrid combinations, are successful under particular environmental conditions, or that only hybrid genotypes are able to spread extensively in new environments. In Australia, *P. pallida* occurs widely in the north of the country, from the east coast of Queensland through the Northern territory, to the west coast of Western Australia (van Klinken and Campbell 2001; CRC Weed Management Guide 2003; van Klinken 2012). However, this species is not found in the cooler southern states of Australia, where *P. velutina* and hybrids between this species and *P. glandulosa* var. *torreyana* seem to dominate (van Klinken and Campbell 2001; CRC Weed Management Guide 2003; van Klinken 2012). While these biogeographic patterns may reflect the initial introduction of only certain species to certain areas (van Klinken and Campbell 2001), they may also be indicative of variation in soil or climate preferences of these species and their hybrids.

Our results also show that polyploidization facilitates immediate reproductive isolation between *Prosopis* species. Polyploidy often also leads to higher levels of stress tolerance, growth vigour through increased plant size, seed size, flower size, niche breadth and phenotypic plasticity, among others, traits that will benefit invasive species (for a review, see te Beest *et al.* 2012). This may well explain why only tetraploid *P. juliflora*, and not diploid *P. pallida*, became invasive in Eastern Africa, despite the similar introduction histories of the two species to the region.

Our findings may also have implications for the management of *Prosopis* invasions. For example, the fact that *P. juliflora* is genetically highly differentiated from other *Prosopis* species raises the question whether biological control agents that have been tested against (mostly diploid) invasive *Prosopis* species in Australia and South Africa could perform differently on invasive *P. juliflora* in Eastern Africa. Moreover, hybridization between *Prosopis* species may also reduce the likelihood of finding effective biological control agents against any particular taxon (Goolsby *et al.* 2006). Lastly, given extensive hybridization between *Prosopis* species, it may be prudent to treat diploid species from the Americas as a single unit when developing regulations to govern management, and not to regulate individual species. This not only has obvious management advantages, but also circumvents potential legal challenges to such regulations (e.g. see Little 2019). However, even under such a classification scheme we think that future research should still aim to determine whether different taxa and their hybrids differ in invasiveness and their responses to different management practices.

## Supporting Information

The following additional information is available in the online version of this article—

Appendix S1. Supporting Information—materials and methods.

Table S1. Details of the 51 microsatellites loci tested for amplification.

Table S2. Volume of the 11 microsatellites primers included in one multiplex PCR assay.

Table S3. List of *Prosopis* individuals, from native and non-native populations, that presented more than two alleles in at least one locus.

Table S4. Number of alleles per microsatellites locus for native and non-native populations of different *Prosopis* species, putative hybrids and *Prosopis* spp. individuals.

Table S5. Pairwise *F*_ST_ values calculated for various native and non-native populations of *Prosopis* species, putative hybrids and *Prosopis* spp. individuals.

Table S6. 95 % confidence interval of pairwise *F*_ST_ values (calculated on bootstrap resampling over loci) for various native and non-native populations of *Prosopis* species, putative hybrids and *Prosopis* spp. individuals.

Table S7. Pairwise G’’ST values calculated for various native and non-native populations of Prosopis species, putative hybrids and Prosopis spp. individuals.

Figure S1. Identification of the optimal number clusters (*K*) inferred by an hierarchical Bayesian clustering analyses with the software STRUCTURE.

Figure S2. Percentage of individuals assigned to genotype classes by NewHybrids software using seven nuclear microsatellites loci.

plaa069_suppl_Supplementary_Materials

## Data Availability

The genotype data generated in the study are available on the DRYAD online repository (doi:10.5061/dryad.zgmsbcc97).

## Notes

**Populations & Communities. Chief Editor:** Jean Burns
