# Supplementary material for: Genetic insights into the globally invasive and taxonomically problematic tree genus Prosopis
Source: AoB Plants. 2020 Dec 8;13(1):plaa069. doi: 10.1093/aobpla/plaa069 (PMC7846124; doi:10.1093/aobpla/plaa069)
Supplement: plaa069_suppl_Supplementary_Materials [file plaa069_suppl_supplementary_materials.pdf]

SUPPORTING INFORMATION

APPENDIX S1

Supplementary information: materials and methods

Sampling and DNA extraction

In the Americas, the genus *Prosopis* is distributed from the south-west of the United States to Argentinean and Chilean Patagonia with two centers of radiation: the Argentine–Paraguayan–Chilean region with 31 species, of which 11 species are considered endemic to Argentina (Burkart 1976; Hunziker *et al.* 1986), and the Texan–Mexican region with 10 species, three of which are endemic (Burkart 1976; Rzedowski 1988). Considering this, our collections from native areas included sites spanning these two centers of radiation of the genus.

With respect to the native range of these species, *P. laevigata* is found across central Mexico, while *P. juliflora* occurs along the Pacific coast from the south of Sonora until Chiapas in Mexico (Rzedowski 1988; Ramírez-Martínez 2015), but also extends its distribution through arid and semi-arid regions of Central and South America to Colombia (Pasiecznik *et al.* 2001). Other species in the genus sampled are restricted to South America. *Prosopis pallida* is distributed in arid and semiarid areas of the Pacific coast from Colombia to Peru (Pasiecznik *et al.* 2001; Palacios *et al.* 2012). *Prosopis alba* occurs in north and central Argentina and the north of Chile. *Prosopis chilensis* is commonly found in parts of north-western Argentina, from the north to the centre of Chile, southern Peru and Bolivia (Burkart 1976). *Prosopis flexuosa* is found in the arid regions of western Argentina and northern Chile (Alvarez and Villagra 2010). *Prosopis nigra* is distributed in the central areas of northern Argentina, in subtropic areas of Paraguay and Uruguay and in arid zones of Bolivia. *Prosopis tamarugo* occurs in the north of Chile. The endemic *P. torcuata* occurs only in northwestern Argentina. *Prosopis strombulifera* is found in areas of western Argentina and northern Chile, while *P. vinalillo* occurs in northern Argentina (FAO 2000).

Species and putative hybrids were provisionally identified using morphological characters and the identification key of Burkart (1976). The latter primarily considers characteristics of leaves (i.e. leave sizes, leave length, leaflet length, space between leaflets, pubescence or glabrous morphology), but also characteristics of seed pods (i.e. shape, length, width) and inflorescences (i.e. raceme length), tree form and the presence of thorns. Genomic DNA extractions from dried leaf tissue were performed following the cetyltrimethylammonium

bromide (CTAB) protocol (Doyle and Doyle 1990). All DNA extractions were diluted to a standard concentration of 50ng/µL and 100 ng/µL for microsatellite and DNA sequencing, respectively. According to previous studies on ploidal variation in *Prosopis*, the only polyploid species in the genus is *P. juliflora* (2n = 4*x* = 46), with all other species being entirely diploid (2n = 2*x*= 28) (Trenchard *et al.* 2008). Considering this, we performed flow cytometry analysis on a subset of the *P. juliflora* sampled individuals (n=63 for Kenya; n=10 for Ethiopia; n=2 for Mexico). Briefly, individuals morphologically identified as *P. juliflora* were mostly tetraploid, but occasionally were identified as triploid individuals (n=17) in Kenya and diploid individuals (n=3) in Kenya and Ethiopia (M.L. Castillo, unpublished data). These individuals were included in the genetic analyses considering their ploidy level (see below).

Nuclear and chloroplast DNA sequencing

For initial optimization of gene amplification and sequencing, we selected one individual of *P. alba*, *P. chilensis* and *P. tamarugo* from Chile; *P flexuosa*, *P. vinalillo* and *P. nigra* form Argentina; *P. laevigata and P. juliflora* from Mexico; *P pallida* from Peru; *P. juliflora* from Kenya, one unidentified individual from South Africa, and one putative hybrid from Australia. For each gene region, polymerase chain reactions (PCRs) were performed in 30 µL reaction volumes, each containing 3 µL of genomic DNA, 0.6 µL of each dNTP (AB gene; Southern Cross Biotechnologies, Cape Town, South Africa), 3 µL of each primer, 10 X PCR reaction buffer, 25 mM $MgCl_2$ and 0.6 µL of BSA (Promega), 0.6 µL *Taq* DNA Polymerase (Super Therm JMR-801; Southern Cross Biotechnologies). The *ETS* region was amplified using the primers described in Brown *et al.* (2008) and PCR cycling included initial denaturation at 95 °C for 3 min, followed by 30 cycles of denaturation at 94 °C for 60s, annealing at 60 °C for 60s, elongation at 72 °C for 2 min; and final extension at 72 °C for 10 min. For the *rpl32-trnL* region we used the primers described by Shaw *et al.* (2007) and PCR cycling of initial denaturation at 95 °C for 2 min, followed by 30 cycles at denaturation at 95 °C for 30s, annealing at 60 °C for 30s, elongation at 72 °C for 60s; and final extension at 72 °C for 10 min. For the *psbA-trnH* region we used the primers described by Sang *et al.* (1997) and a PCR cycle of an initial denaturation at 80 °C for 5 min, followed by 30 cycles at denaturation at 94 °C for 30s, annealing at 60 °C for 30s, elongation at 72 °C for 60s; and final extension at 72 °C for 10 min. Amplified DNA fragments were purified using the Qiagen PCR Purification kit (supplied by Whitehead Scientific, Cape Town, South Africa) and sequenced using the ABI PRISM BigDye Terminator Cycle Sequencing Ready Reaction kit and an automated ABI PRISM 377XL DNA sequencer (PE Applied Biosystems, Foster City, CA, USA).

Microsatellite genotyping

We tested cross-amplification success for 51 microsatellite loci previously developed and characterized in numerous *Prosopis* species [see Table S1]. These microsatellite primers were tested under different temperature conditions and we were able to obtain successful amplification for 30 loci. Amplification of these markers was performed using multiplex PCR assays for which markers with non-overlapping amplicon size were combined. Each multiplex PCR reaction contained 1.5 µL of primer mix, 7.5 µL of KAPA2G Fast Multiplex Mix (Kapa Biosystem, Cape Town, South Africa), 4.5 µL purified H_2O and 1.5 µL of DNA in a total reaction volume of 15 µL. The volume of each primer in the primer mix is provided in Supporting Information—Table S2. The PCR cycle included 3 min denaturation at 95 °C, followed by 30 cycles of denaturation at 95 °C for 15 s, annealing at 60 °C for 30 s, elongation at 72 °C for 25 min, and a final elongation at 72 °C for 1 min. PCR reactions were performed in 96-well plates containing 89 DNA samples and five randomly selected technical replicates and two negative controls (H_2O). Amplified fragments were submitted for gel capillary electrophoresis at Stellenbosch University's Central Analytical Facility. Automated allele scoring was done using the GeneMarker version 2.6.4 software (SoftGenetics LLC, Pennsylvania, United States) and all alleles were manually checked.

Genetic structure and hybridization

A hierarchical clustering approach (Le Roux *et al.* 2010) was implemented using the software STRUCTURE. This software uses Bayesian Monte–Carlo Markov chain sampling to identify the optimal number of genetic clusters for a given dataset by reducing departures from Hardy–Weinberg and linkage equilibrium expectations within genetic clusters. For the first level clustering, a number of genetic clusters (K) between K=1 and K=12 (number of species sampled and that amplified successfully), were tested. For each value of K that were run, we tested 10 independent models. Each model consisted of 500,000 generations of which the first 100,000 were discarded as burnin. An admixture model with correlated allele frequencies was applied since this type of model is more robust for identifying the optimal number of clusters that captures the major structure of the data (François and Durand 2010) and enables the identification of hybrids. STRUCTURE provides assignment values for each individual to each identified genetic cluster, calculated as the proportion (q_{ik}) of each genotype individuals sampled that is derived from each of the K clusters. Individuals not assigned to a single genetic cluster with $q_{ik} > 0.6$ were excluded from subsequent analyses. Assignment values were also

used to determine the presence of hybrids, with individuals having similar assignment values (*qik*) to each genetic cluster(s) being classified as hybrids. The analysis excluded all non-*P. juliflora* polyploids (i.e. individuals of other species that had more than two alleles at loci, n=14; Supporting Information—Table S3). Since the dataset included individuals of different ploidy levels, an overall ploidy of 4*x* was specified in the analyses. For analyses including *P. juliflora* polyploid individuals, the option *RECESSIVEALLELES* was set to one to account for allele copy ambiguity (Pritchard *et al.* 2010). For diploid-triploid individuals, a missing data symbol was added to complete the ploidal level, indicating that the individual is diploid-triploid at all loci (Pritchard *et al.* 2000). The optimal *K* value was estimated following the method described by Evanno *et al.* (2005) and STRUCTURE Harvester (Earl and vonHoldt 2012). CLUMPAK software (Kopelman *et al.* 2015) was used to graphically display the results.

Table S1. Details of the 51 microsatellites loci tested for amplification. From these, 11 markers were included in one multiplex PCR assay (in bold).

Locus name	Original study	Species for which microsatellites were originally developed
Prb1	Alves *et al.* 2014	*P. rubriflora*
Prb2	Alves *et al.* 2014	*P. rubriflora*
Prb3	Alves *et al.* 2014	*P. rubriflora*
**Prb4**	Alves *et al.* 2014	*P. rubriflora*
Prb5	Alves *et al.* 2014	*P. rubriflora*
Prb6	Alves *et al.* 2014	*P. rubriflora*
Prb7	Alves *et al.* 2014	*P. rubriflora*
**Prb8**	Alves *et al.* 2014	*P. rubriflora*
Prb9	Alves *et al.* 2014	*P. rubriflora*
Prb10	Alves *et al.* 2014	*P. rubriflora*
Prsc1	Alves *et al.* 2014	*P. ruscifolia*
Prsc2	Alves *et al.* 2014	*P. ruscifolia*
Prsc3	Alves *et al.* 2014	*P. ruscifolia*
Prsc4	Alves *et al.* 2014	*P. ruscifolia*
Prsc5	Alves *et al.* 2014	*P. ruscifolia*
Prsc6	Alves *et al.* 2014	*P. ruscifolia*
**Prsc7**	Alves *et al.* 2014	*P. ruscifolia*
Prsc8	Alves *et al.* 2014	*P. ruscifolia*
**Prsc9**	Alves *et al.* 2014	*P. ruscifolia*
Prsc10	Alves *et al.* 2014	*P. ruscifolia*
Prsc11	Alves *et al.* 2014	*P. ruscifolia*
Prsc12	Alves *et al.* 2014	*P. ruscifolia*
Prsc13	Alves *et al.* 2014	*P. ruscifolia*
GL6	Bessega *et al.* 2013	*P. alba – P. chilensis*
GL8	Bessega *et al.* 2013	*P. alba – P. chilensis*
GL9	Bessega *et al.* 2013	*P. alba – P. chilensis*
**GL12**	Bessega *et al.* 2013	*P. alba – P. chilensis*
GL15	Bessega *et al.* 2013	*P. alba – P. chilensis*
GL16	Bessega *et al.* 2013	*P. alba – P. chilensis*
GL18	Bessega *et al.* 2013	*P. alba – P. chilensis*
GL21	Bessega *et al.* 2013	*P. alba – P. chilensis*
**GL23**	Bessega *et al.* 2013	*P. alba – P. chilensis*
GL24	Bessega *et al.* 2013	*P. alba – P. chilensis*
GL26	Bessega *et al.* 2013	*P. alba – P. chilensis*
Mo05	Mottura *et al.* 2005	*P. flexuosa – P. chilensis*
Mo07	Mottura *et al.* 2005	*P. flexuosa – P. chilensis*
Mo08	Mottura *et al.* 2005	*P. flexuosa – P. chilensis*

Mo09	Mottura *et al.* 2005	*P. flexuosa – P. chilensis*
Mo13	Mottura *et al.* 2005	*P. flexuosa – P. chilensis*
Mo16	Mottura *et al.* 2005	*P. flexuosa – P. chilensis*
I-P00930b*	Torales *et al.* 2013	*P. alba*
**I-P00930c***	Torales *et al.* 2003	*P. alba*
I-P00930d*	Torales *et al.* 2003	*P. alba*
I-P03211*	Torales *et al.* 2003	*P. alba*
I-P03325a*	Torales *et al.* 2003	*P. alba*
I-P06286b*	Torales *et al.* 2003	*P. alba*
**I-P06639***	Torales *et al.* 2003	*P. alba*
**I-P07653***	Torales *et al.* 2003	*P. alba*
I-P10500*	Torales *et al.* 2003	*P. alba*
**S-P1DKSFA***	Torales *et al.* 2003	*P. alba*
**S-P1EPIV2***	Torales *et al.* 2003	*P. alba*

*Markers with functional annotations

Table S2. Volumes of the 11 microsatellites primers (2µM) used in 50 µL of primer mix. Microsatellites were included in one multiplex PCR assay. From these, seven were included in the study (in bold).

Locus name	Volume primer (µl)
**Prb4**	1
Prb8	2
**Prsc7**	1
**Prsc9**	0.5
**GL12**	1
GL23	2
I-P00930c	0.5
**I-P06639**	1
I-P07653	0.3
**S-P1DKSFA**	0.5
**S-P1EPIV2**	1
10 X Buffer	28.4
Total volume	50µl

Table S3. List of *Prosopis* individuals from native and non-native populations that had more than two alleles at at least one locus. These individuals were excluded from subsequent analyses. Non-native range countries are indicated by asterisks (*).

**Species**	**Country**	**Number of individuals**
P. flexuosa	Argentina	1
P. laevigata	Mexico	1
Hybrid	Australia*	1
Prosopis spp.	Argentina	2
Prosopis spp.	South Africa*	9

Table S4. Number of alleles per microsatellites locus for native and non-native populations of different *Prosopis* species, putative hybrids and *Prosopis* spp. individuals from South Africa. Native *Prosopis* and non-native *Prosopis* groups (i.e. all native and non-native *Prosopis* individuals, respectively), were analysed as well. Non-native range countries are indicated by asterisks (*).

**Locus name**	***P. alba***		***P. chilensis***		***P. flexuosa***	***P. juliflora***				***P. laevigata***	***P. nigra***	***P. pallida***			***P. vinalillo***	**Hybrids**		***Prosopis* spp.**	**Native *Prosopis***	**Non-native *Prosopis***
	**Ar**	**Ch**	**Ar**	**Ch**	**Ar**	**Me**	**Et***	**Ke***	**Tz***	**Me**	**Ar**	**Pe**	**Hw***	**Ke***	**Ar**	**Ar**	**Au***	**SA***		
GL12	13	8	9	8	6	2	4	8	4	6	7	5	2	5	10	10	3	13	24	16
I-P06639	4	3	4	4	4	3	2	5	1	3	1	2	1	4	3	2	2	4	8	6
Prb4	18	6	11	11	9	3	7	14	4	11	6	9	6	9	9	10	4	16	27	21
Prsc7	17	11	12	13	9	2	7	8	7	9	6	5	4	7	10	10	3	19	32	24
Prsc9	4	6	4	4	4	3	4	8	7	8	3	1	2	5	3	3	3	8	13	9
S-P1DKSFA	4	3	4	3	2	4	2	5	3	2	2	1	1	2	2	4	1	3	6	3
S-P1EPIV2	6	4	4	6	4	1	2	3	2	2	2	1	1	2	5	5	2	4	6	4
**Overall**	**66**	**41**	**48**	**49**	**38**	**18**	**28**	**51**	**28**	**41**	**27**	**24**	**17**	**34**	**42**	**44**	**18**	**67**	**116**	**83**

Country codes: Ar=Argentina; Au=Australia; Ch=Chile; Et=Ethiopia, Hw=Hawaii; Ke=Kenya, Me=Mexico, Pe=Peru; SA=South Africa; Tz=Tanzania.

Table S5. Pairwise F_{ST} values for various native and non-native populations of *Prosopis* species, putative hybrids and *Prosopis* spp. individuals from South Africa. Non-native range countries are indicated by asterisks (*).

		P. alba		***P. chilensis***		***P. flexuosa***	***P. laevigata***	***P. nigra***	***P. pallida***			***P. vinalillo***	**Hybrids**	
		**Ar**	**Ch**	**Ar**	**Ch**	**Ar**	**Me**	**Ar**	**Pe**	**Hw***	**Ke***	**Ar**	**Ar**	**Au***
P. alba	**Ch**	0.05	-	-	-	-	-	-	-	-	-	-	-	-
P. chilensis	**Ar**	0.01	0.06	-	-	-	-	-	-	-	-	-	-	-
	**Ch**	0.04	0.08	0.03	-	-	-	-	-	-	-	-	-	-
P. flexuosa	**Ar**	0.07	0.09	0.10	0.11	-	-	-	-	-	-	-	-	-
P. laevigata	**Me**	0.17	0.19	0.22	0.23	0.14	-	-	-	-	-	-	-	-
P. nigra	**Ar**	0.13	0.12	0.16	0.16	0.02	0.11	-	-	-	-	-	-	-
P. pallida	**Pe**	0.24	0.36	0.28	0.30	0.31	0.45	0.40	-	-	-	-	-	-
	**Hw***	0.31	0.44	0.34	0.35	0.40	0.48	0.49	0.15	-	-	-	-	-
	**Ke***	0.31	0.43	0.33	0.34	0.37	0.46	0.40	0.12	0.08	-	-	-	-
P. vinalillo	**Ar**	0.05	0.07	0.07	0.10	-0.03	0.13	0.00	0.32	0.40	0.35	-	-	-
**Hybrids**	**Ar**	0.03	0.03	0.03	0.04	0.04	0.13	0.05	0.31	0.37	0.34	-0.01	-	-
	**Au***	0.07	0.15	0.11	0.15	0.05	0.16	0.12	0.25	0.35	0.26	0.09	0.11	-
***Prosopis* spp.**	**SA***	0.06	0.07	0.08	0.07	0.08	0.13	0.09	0.26	0.28	0.28	0.06	0.04	0.09

Country codes: Ar=Argentina; Au=Australia; Ch=Chile; Et=Ethiopia, Hw=Hawaii; Ke=Kenya, Me=Mexico, Pe=Peru; SA=South Africa; Tz=Tanzania.

Table S6. 95% confidence intervals of pairwise F_{ST} values (calculated via bootstrap resampling across loci) for various native and non-native populations of *Prosopis* species, putative hybrids and *Prosopis* spp. individuals from South Africa. Non-native range countries are indicated by asterisks (*).

		P. alba		***P. chilensis***		***P. flexuosa***	***P. laevigata***	***P. nigra***	***P. pallida***			***P. vinalillo***	**Hybrids**	
		**Ar**	**Ch**	**Ar**	**Ch**	**Ar**	**Me**	**Ar**	**Pe**	**Hw***	**Ke***	**Ar**	**Ar**	**Au***
P. alba	**Ch**	0.10-0.02	-	-	-	-	-	-	-	-	-	-	-	-
P. chilensis	**Ar**	0.03--0.01	0.03-0.00	-	-	-	-	-	-	-	-	-	-	-
	**Ch**	0.06-0.01	0.06-0.02	0.04-0.01	-	-	-	-	-	-	-	-	-	-
P. flexuosa	**Ar**	0.15-0.00	0.13-0.01	0.20-0.02	0.17-0.04	-	-	-	-	-	-	-	-	-
P. laevigata	**Me**	0.29-0.07	0.25-0.06	0.34-0.08	0.30-0.10	0.25-0.04	-	-	-	-	-	-	-	-
P. nigra	**Ar**	0.25-0.01	0.20-0.01	0.29-0.05	0.25-0.06	0.06--0.01	0.21-0.00	-	-	-	-	-	-	-
P. pallida	**Pe**	0.45-0.09	0.44-0.10	0.50-0.10	0.46-0.13	0.56-0.08	0.64-0.21	0.68-0.11	-	-	-	-	-	-
	**Hw***	0.46-0.17	0.45-0.18	0.52-0.19	0.46-0.18	0.60-0.20	0.65-0.24	0.72-0.20	0.31-0.04	-	-	-	-	-
	**Ke***	0.44-0.19	0.44-0.19	0.48-0.19	0.44-0.19	0.57-0.17	0.64-0.24	0.66-0.16	0.24-0.02	0.13-0.02		-	-	-
P. vinalillo	**Ar**	0.12-0.00	0.09-0.00	0.15-0.02	0.14-0.03	-0.01--0.05	0.24-0.03	0.03--0.03	0.58-0.09	0.61-0.18	0.60-0.12	-	-	-
**Hybrids**	**Ar**	0.06-0.00	0.04-0.00	0.06-0.00	0.06-0.00	0.07-0.01	0.22-0.06	0.08-0.01	0.53-0.14	0.55-0.20	0.57-0.15	0.04--0.04	-	-
	**Au***	0.13-0.01	0.13-0.03	0.19-0.03	0.16-0.07	0.09--0.02	0.29-0.06	0.27-0.02	0.52-0.03	0.61-0.16	0.51-0.09	0.16-0.01	0.14-0.07	-
***Prosopis* spp.**	**SA***	0.08-0.04	0.07-0.03	0.10-0.05	0.09-0.04	0.15-0.03	0.18-0.08	0.13-0.03	0.41-0.13	0.41-0.16	0.43-0.15	0.12-0.01	0.07-0.01	0.11-0.07

Country codes: Ar=Argentina; Au=Australia; Ch=Chile; Et=Ethiopia, Hw=Hawaii; Ke=Kenya, Me=Mexico, Pe=Peru; SA=South Africa; Tz=Tanzania.

Table S7. Pairwise G''_{ST} values calculated for various native and non-native populations of *Prosopis* species, putative hybrids and *Prosopis* spp. individuals from South Africa. Non-native range countries are indicated by asterisks (*).

		P. alba		***P. chilensis***		***P. flexuosa***	***P. juliflora***				***P. laevigata***	***P. nigra***	***P. pallida***			***P. vinalillo***	**Hybrids**	
		**Ar**	**Ch**	**Ar**	**Ch**	**Ar**	**Me**	**Et***	**Ke***	**Tz***	**Me**	**Ar**	**Pe**	**Hw***	**Ke***	**Ar**	**Ar**	**Au***
P. alba	**Ch**	0.18	-	-	-	-	-	-	-	-	-	-	-	-	-	-	-	-
P. chilensis	**Ar**	0.04	0.20	-	-	-	-	-	-	-	-	-	-	-	-	-	-	-
	**Ch**	0.14	0.26	0.09	-	-	-	-	-	-	-	-	-	-	-	-	-	-
P. flexuosa	**Ar**	0.22	0.30	0.33	0.34	-	-	-	-	-	-	-	-	-	-	-	-	-
P. juliflora	**Me**	0.78	0.82	0.83	0.83	0.69	-	-	-	-	-	-	-	-	-	-	-	-
	**Et***	0.78	0.82	0.83	0.83	0.69	0.46	-	-	-	-	-	-	-	-	-	-	
	**Ke***	0.74	0.80	0.79	0.79	0.67	0.50	0.03	-	-	-	-	-	-	-	-	-	-
	**Tz***	0.83	0.87	0.87	0.88	0.78	0.69	0.26	0.27	-	-	-	-	-	-	-	-	-
P. laevigata	**Me**	0.43	0.39	0.50	0.52	0.30	0.70	0.77	0.76	0.85	-	-	-	-	-	-	-	-
P. nigra	**Ar**	0.35	0.31	0.41	0.42	0.04	0.79	0.77	0.74	0.85	0.21	-	-	-	-	-	-	-
P. pallida	**Pe**	0.58	0.75	0.63	0.66	0.66	0.87	0.88	0.83	0.90	0.78	0.70	-	-	-	-	-	-
	**Hw***	0.65	0.78	0.68	0.68	0.69	0.88	0.90	0.85	0.92	0.76	0.71	0.20	-	-	-	-	-
	**Ke***	0.60	0.75	0.62	0.62	0.65	0.86	0.86	0.81	0.90	0.73	0.67	0.18	0.15	-	-	-	-
P. vinalillo	**Ar**	0.17	0.21	0.23	0.30	-0.10	0.74	0.73	0.70	0.81	0.28	0.00	0.66	0.68	0.61	-	-	-
**Hybrids**	**Ar**	0.11	0.12	0.10	0.13	0.12	0.79	0.81	0.78	0.88	0.28	0.12	0.70	0.68	0.62	-0.01	-	-
	**Au***	0.21	0.43	0.32	0.40	0.14	0.57	0.57	0.51	0.73	0.29	0.27	0.43	0.48	0.40	0.26	0.31	-
***Prosopis* spp.**	**SA***	0.19	0.22	0.25	0.21	0.25	0.72	0.70	0.67	0.78	0.32	0.23	0.62	0.62	0.56	0.19	0.14	0.27

Country codes: Ar=Argentina; Au=Australia; Ch=Chile; Et=Ethiopia, Hw=Hawaii; Ke=Kenya, Me=Mexico, Pe=Peru; SA=South Africa; Tz=Tanzania.

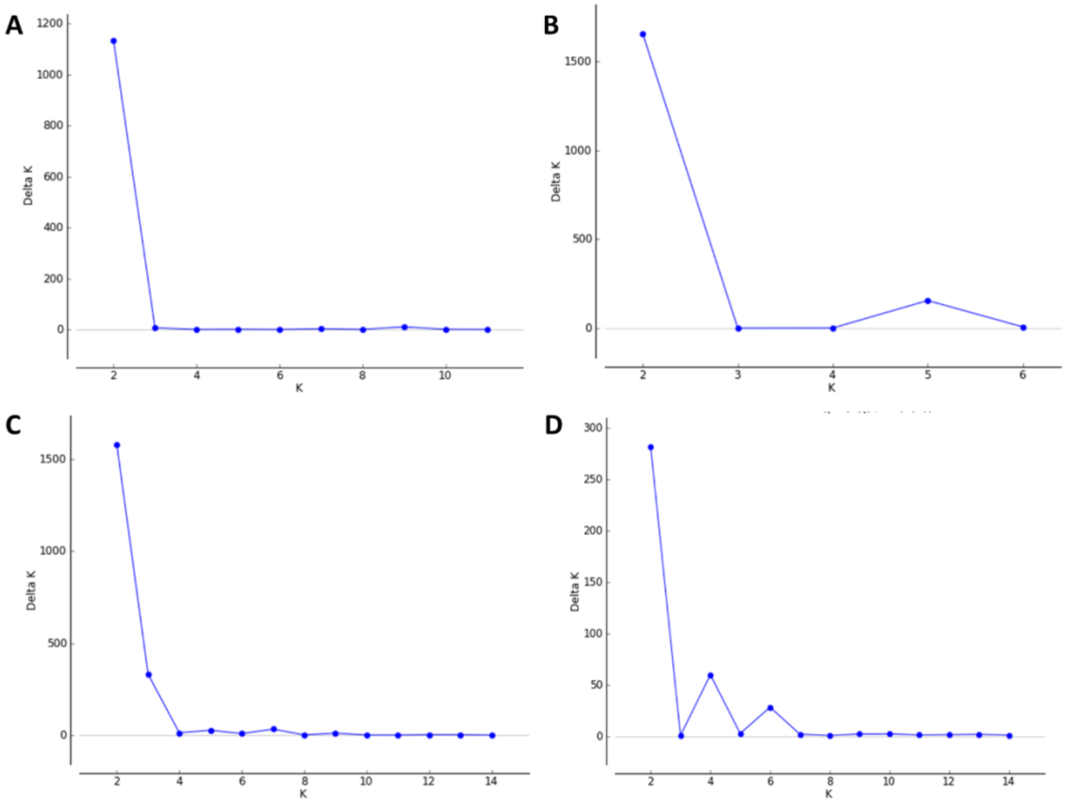

Figure S1. Identification of the optimal number clusters (K) inferred by an hierarchical Bayesian clustering analyses with the software STRUCTURE. The level of clustering includes: (A) Level 1, with all *Prosopis* individuals representing 11 species; Level 2, with (B) only *P. juliflora* individuals from Mexico, Ethiopia, Kenya and Tanzania, and (C) all diploid *Prosopis* species (i.e. excluding *P. juliflora*); (D) Level 3, including only *Prosopis* species from Argentina, Australia, Chile, Mexico, and South Africa. In all cases *K*=2 were identified as the optimal number of genetic clusters. Individuals were genotyped using seven nuclear microsatellites loci (see Material and Methods for model parameters).

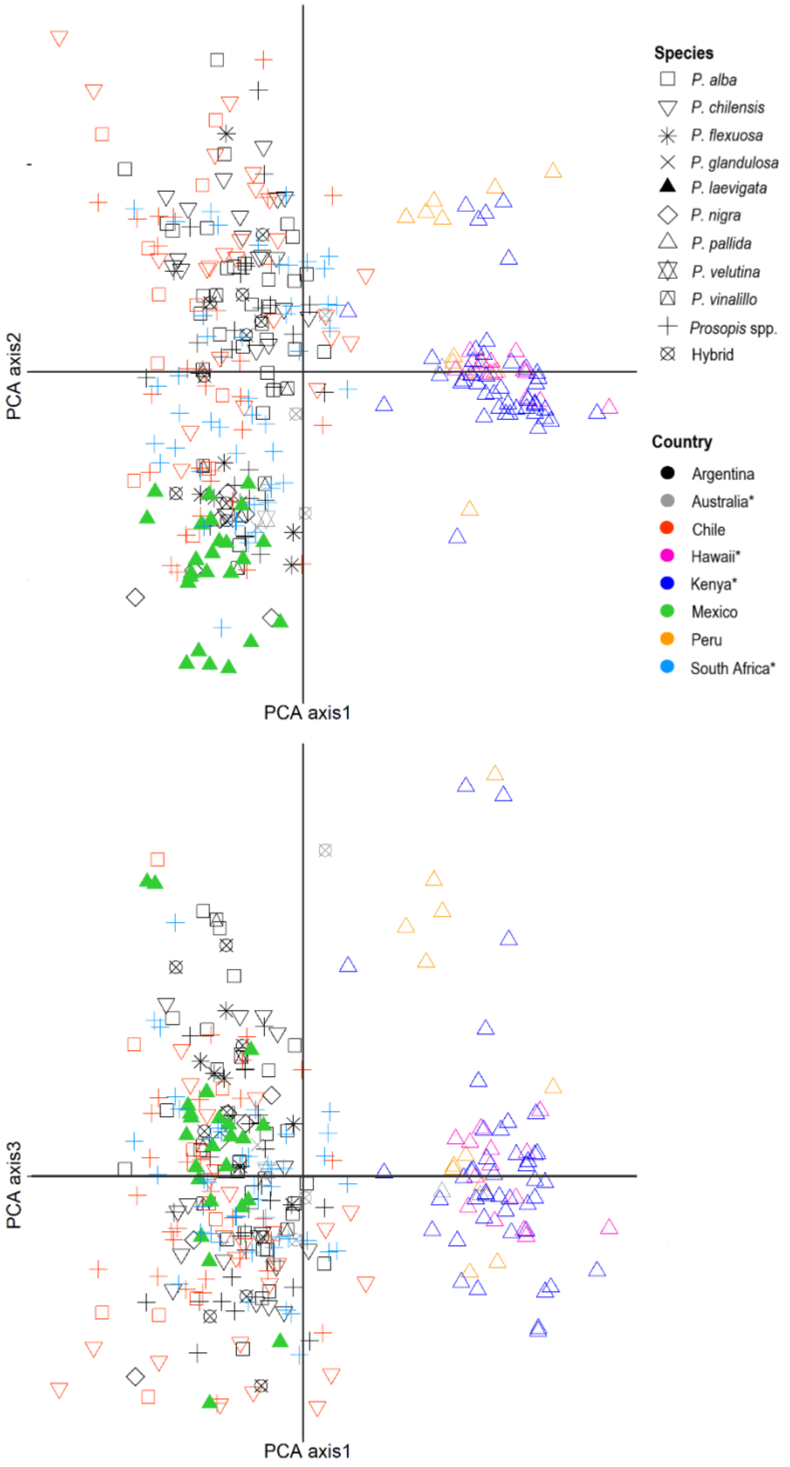

Figure S2. '*Diploid-only'* principal component analysis (PCA) showing genetic structure among native and non-native populations of different diploid *Prosopis* species, putative hybrids and *Prosopis* spp. individuals. Non-native range countries are indicated by asterisks (*). PCA was performed using Euclidian distances between individuals. PCA 1, PCA 2 and PCA 3 captured 25.5%, 10.4% and 5.5% of the variation, respectively.

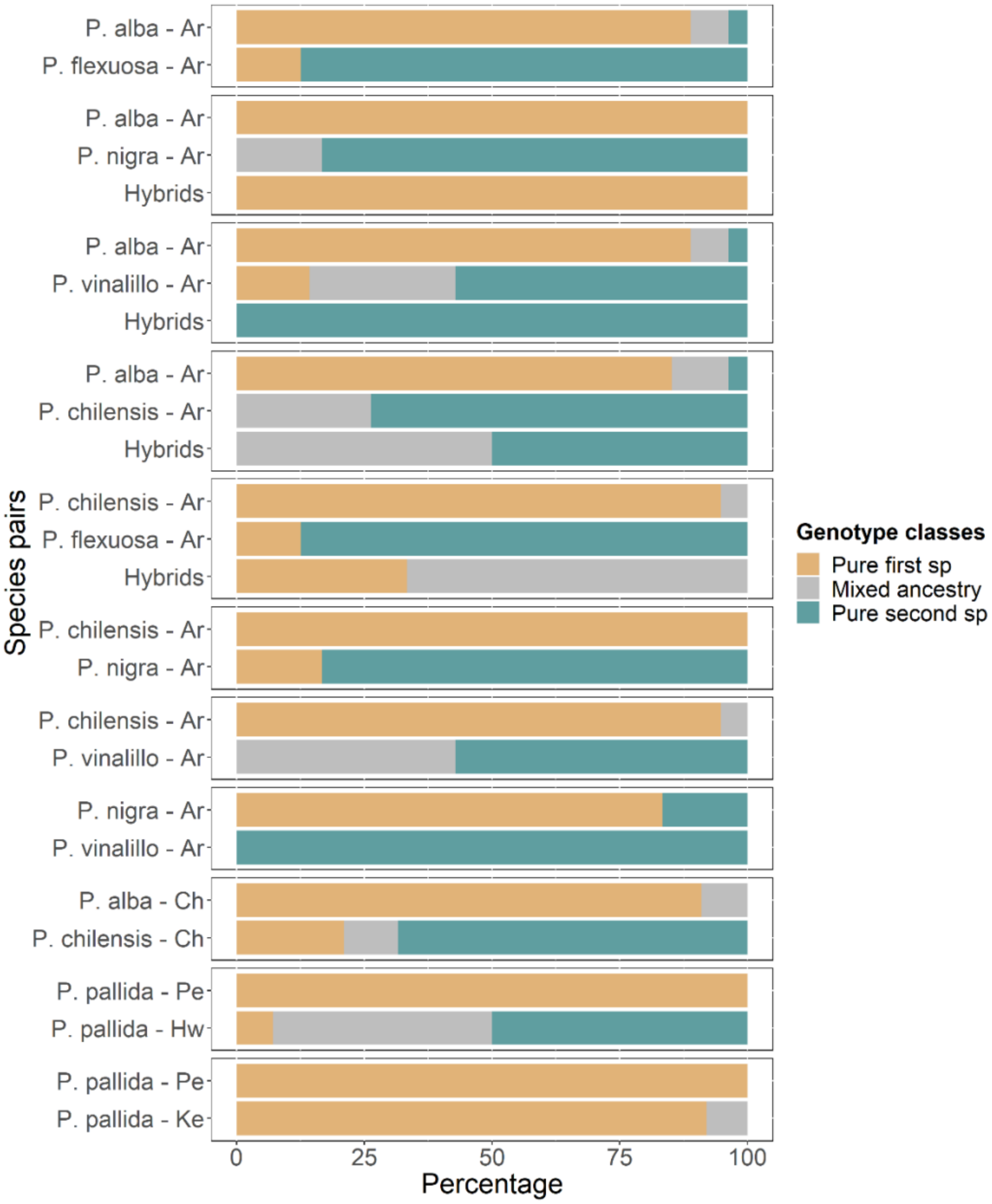

Figure S3. Percentage of individuals assigned to genotype classes: pure parental species 1, pure parental species 2, or mixed ancestry; by NewHybrids software using data from seven nuclear microsatellite loci. The analysis was done on pairs of *Prosopis* species and hybrids identified morphologically from various native areas: Argentina (Ar), Chile (Ch) and Peru (Pe). Native *P. pallida* individuals from Peru (Pe) were compared with introduced individuals in Kenya (Ke) and invasive individuals in Hawaii (Hw), respectively (see Material and Methods for model parameters).
